# Do incretins improve endothelial function?

**DOI:** 10.1186/1475-2840-13-21

**Published:** 2014-01-15

**Authors:** Jun-ichi Oyama, Yukihito Higashi, Koichi Node

**Affiliations:** 1 Department of Cardiovascular Medicine, Saga University, 5-1-1 Nabeshima, Saga 849-8501, Japan; 2 Department of Cardiovascular Regeneration and Medicine, Research Institute for Radiation Biology and Medicine, Hiroshima University, Hiroshima, Japan

**Keywords:** Incretin, GLP-1, DPP-4 inhibitor, Endothelial function, Diabetes mellitus type 2

## Abstract

An impaired endothelial function has been recognized in the early stage of atherosclerosis, and is a major factor affecting the future development of cardiovascular events. Type 2 diabetes mellitus (T2DM) is widely prevalent, and is one of the most important risk factors for cardiovascular disease. T2DM is associated with increases in both morbidity and mortality, particularly from cardiovascular disease.

New therapies based on the incretin hormone and its actions are now becoming widely used, and appear to offer advantages over conventional therapies by keeping the body weight steady and limiting hypoglycemia, while also achieving attractive glycemic control. However, there is little data available about the effects of incretins on the cardiovascular system.

This review will focus on the effects of incretin therapies, including glucagon-like peptide-1 (GLP-1) analogs and dipeptidyl peptidase (DPP)-4 inhibitors, on the endothelial function, and will discuss the potential mechanisms underlying these effects.

## Introduction

Type 2 diabetes (T2DM) is one of the most important risk factors for the development of cardiovascular disease (CVD) because it promotes systemic atherosclerosis and lifestyle-associated diseases. Recently, increasing evidence has revealed that impaired endothelial function leads to future cardiovascular events, including those in patients with T2DM. Endothelial dysfunction is an early feature of atherosclerosis that is characterized by a reduction of the bioavailability of vasodilators, particularly nitric oxide (NO) [1]. This condition is seen in various clinical populations, including patients with T2DM. The measurement of endothelial function as indicated by the flow-mediated dilation (FMD) via ultrasound has been established as a reliable non-invasive measurement of the endothelial function [2] and has been shown to correlate with the findings of more invasive testing of the endothelial function [3]. Numerous interventions that improve cardiovascular risk factors and reduce cardiovascular morbidity and mortality have been shown to increase the brachial artery reactivity measured by FMD, thus improving the endothelial function [4].

Incretin hormones depend on the level of blood glucose to stimulate insulin. Recently, increasing evidence has suggested that glucagon-like peptide-1 (GLP-1)-related therapy has potent pleiotropic benefits on cardiovascular risk factors, beyondthe glycemic control. This review focuses on the theoretical and practical effects of incretin-related therapy on endothelial function, and describes the possible mechanism(s) of action.

### Biology of incretin hormones

**Figure 1:**
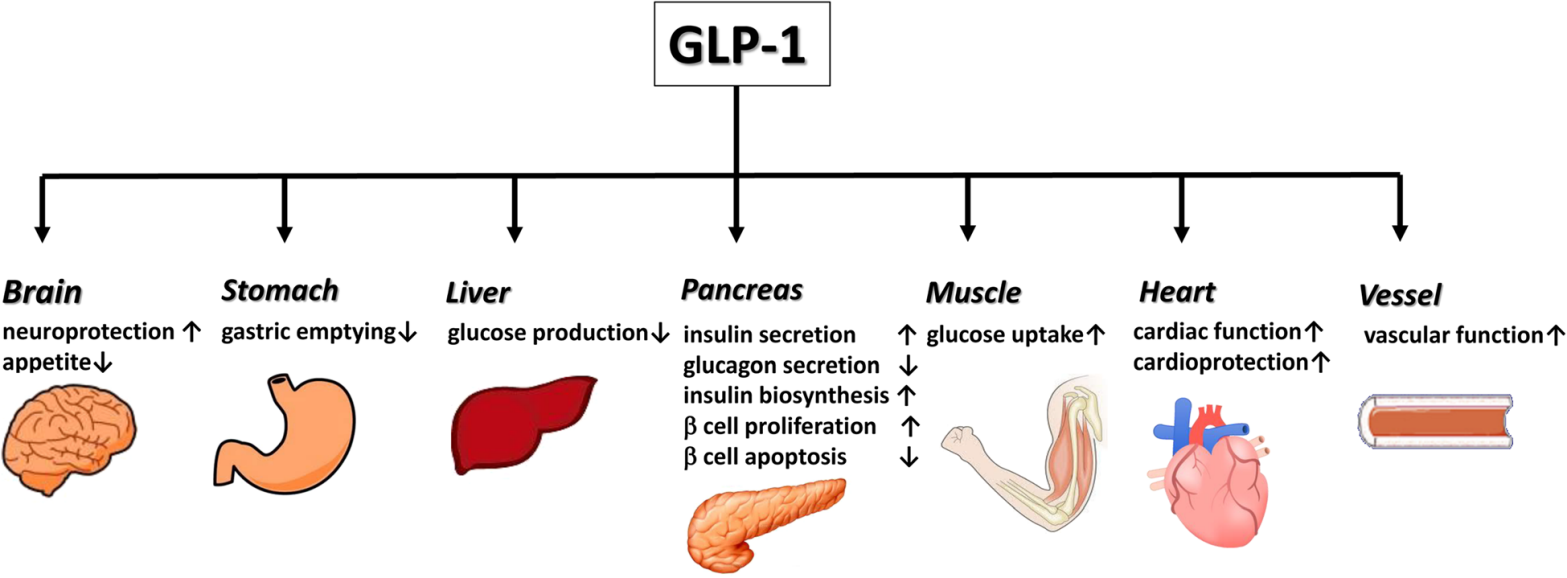
Pleiotropic effects of GLP-1.

Incretin hormones are secreted from the gastrointestinal tract in response to food intake and have several systemic effects, including the glucose-dependent stimulation of insulin secretion by pancreatic beta-cells. Two incretins have been identified: GLP-1, derived from the L-cells of the distal small intestine and large bowel, and glucose-dependent insulinotropic polypeptide (GIP), derived from the K-cells of the proximal small intestine. GLP-1 and GIP are glucose-lowering agents that can interfere with postprandial hyperglycemia, which has been demonstrated to be associated with cardiovascular complications. GLP-1 is secreted primarily in two forms, GLP-1-(7–37) and GLP-1-(7–36)NH(2), both of which bind to a specific GLP-1 receptor (GLP-1R) on the pancreatic β-cell and augment glucose-stimulated insulin secretion. These peptides arise from the selective cleavage of the proglucagon molecule. GLP-1(7–36) amide is abundant in the circulation after meals and stimulates insulin secretion by interacting with GLP-1R on pancreatic β-cells. Activation of GLP-1R on β-cells leads to rapid increases in levels of cAMP and intracellular calcium followed by insulin exocytosis in a glucose-dependent manner [5]. GLP-1R, a G protein–coupled receptor, has been detected in the nervous system, heart, vascular smooth muscle cells, endothelial cells, monocytes and macrophages as well as the gastrointestinal tract [6] (Figure 1).

**Figure 2:**
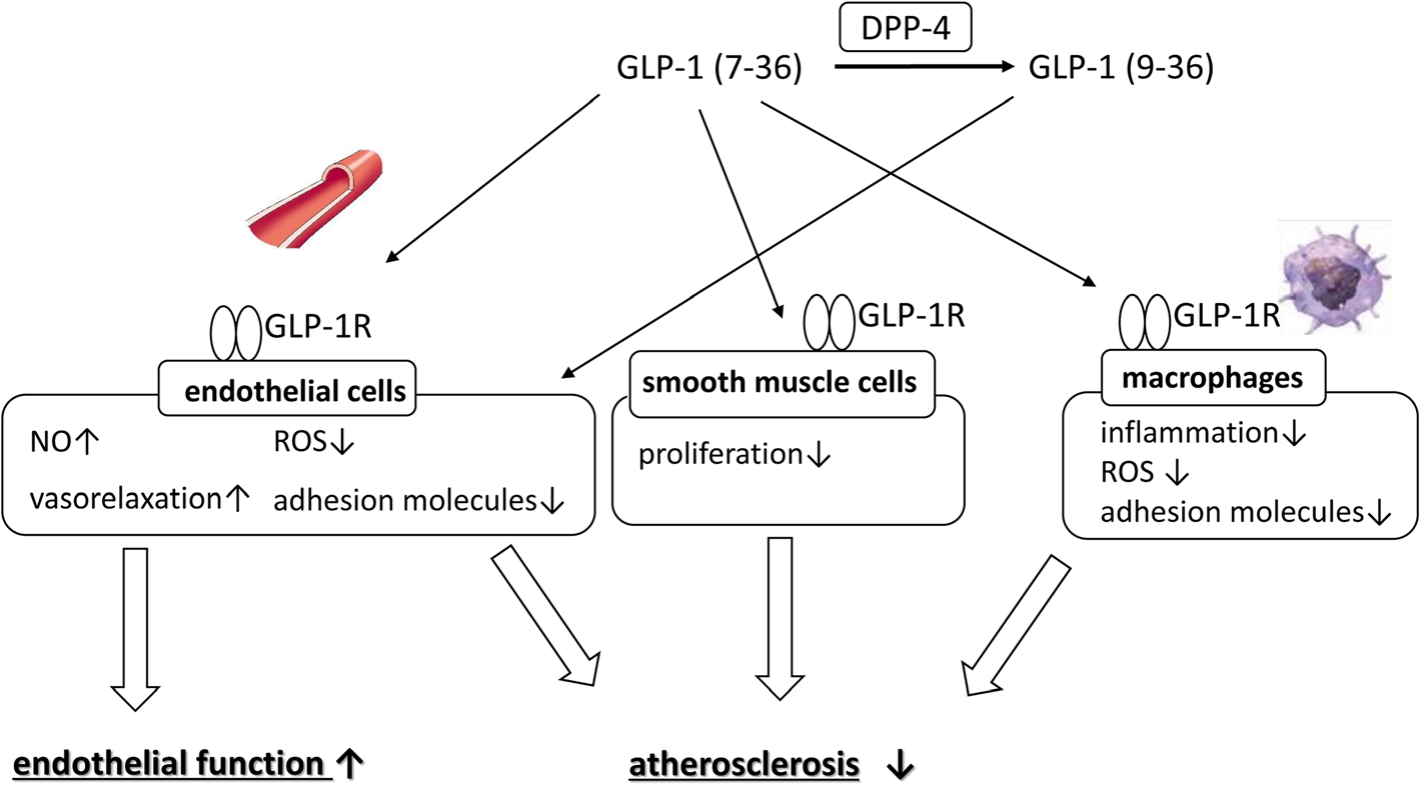
Beneficial effects of GLP-1 on vascular system.

The enzyme dipeptidyl peptidase (DPP)-4, also known as adenosine deaminase complexing protein 2, degrades GLP-1 to inactive GLP-1(9–36), and DPP-4 inhibitors bind to DPP-4 to prevent the breakdown of GLP-1 and GIP [7], thus increasing the half-life and bioavailability of active incretins, and enhancing their physiological effects. GLP-1(7–36) amide has been widely studied for its role as an active incretin and is referred to as GLP-1, unless otherwise specified. GLP-1(9–36) is thought to be an inactive metabolite due to its 1,000-fold lower affinity for GLP-1R and actions as a weak competitive antagonist without an incretin activity at pharmacological doses. However, GLP-1(9–36) may have potent effects on the cardiovascular system, similar to GLP-1(7–36) amide (Figure 2). Although it remains controversial, GLP-1 may undergo multiple steps of enzymatic degradation by DPP-4 and neutral endopeptidase.

### Incretin-related drugs

Nowadays, incretin-based therapies, including GLP-1R agonists and DPP-4 inhibitors, are becoming widely used as a new class of anti-diabetic drugs that exhibit different mechanisms of action from the conventional anti-diabetic drugs (Table 1).

**Table 1:** Features of incretin-related therapy

	**Administration**	**Clearance**	**Side effects**	
GLP-1R agonist	Subctaneous		Weight loss	Gastrointestinal tract (GIT)
Exenatide	Twice a day, once a week	Renal	+	++/±*
Liraglutide	Once a day	Extrarenal	+	+
DPP-4 inhibitor	Oral		→	→
Sitagliptin	Once a day	Renal	→	→
Vildagliptin	Twice a day	Renal	→	→
Saxagliptin	Once a day	Renal	→	→
Linagliptin	Once a day	Extrarenal	→	→
Anagliptin	Twice a day	Renal	→	→
Teneligliptin	Once a day	Extrarenal > renal	→	→
Alogliptin	Once a day	Renal	→	→

*Exenatide once weekly has less side effects of GIT as compared to linagrutide.

Exenatide, one of the GLP-1R agonists and incretin mimetics, bears a 50% amino acid homology to human GLP-1 and it has a longer half-life in vivo, however, has its side effects including weight loss, nausea and vomiting. It must be injected twice daily. Liraglutide, a once-daily GLP-1 derivative, is also a long-acting GLP-1R agonist that shares 97% sequence identity to human GLP-1(7–37) and has a plasma half-life of 13 hours after subcutaneous administration in contrast to a short half-life of native GLP-1. DPP-4 inhibitors are also available with fewer side effects. ADA/EASD/IDF statement concerning the use of incretin therapy and pancreatic disease was reported in June, 2013. The strong relationship between incretin-therapy and pancretitis/pancreatic cancer was not recognized for now, however, we need to look at carefully.

Since DPP-4 inhibitors are associated with a lower incidence of hypoglycemia compared to conventional hypoglycemic drugs, they have been suggested to improve the mortality of patients with T2DM because they can enforce strict glycemic control without causing fatal hypoglycemia.

### Possible effects of incretins on vessels

#### Incretin and endothelial cells (in vitro)

GLP-1 was shown to attenuate the tumor necrosis factor-alpha (TNF-α)-mediated induction of plasminogen activator inhibitor-1 expression [8] and inhibited advanced glycation end product (AGE)-induced upregulation of vascular cell adhesion molecule (VCAM)-1 in cultured human umbilical vein endothelial cells (HUVEC) [9]. It also upregulated the activity and protein expression of endothelial NO synthase (eNOS) in HUVEC through GLP-1R-dependent (GLP-1 (7–36)-related) and-independent (GLP-1(9–36)-related) pathways [10]. GLP-1 prevented reactive oxygen species (ROS)-induced cell senescence through the activation of protein kinase A in HUVEC [11]. It also decreased high glucose-induced ROS production and decreased the apoptotic index, as well the levels of NADPH oxidase and Rho-expression, with increases in the cAMP/PKA activity in cardiac microvascular endothelial cells [12].

In turn, GLP-1 promoted angiogenesis in a dose-dependent manner, which was decreased by Akt inhibitor IV, a PKC inhibitor and src inhibitor I in a 3D culture system where spherules of HUVEC were embedded in a collagen scaffold [13]. Moreover, GLP-1 restored the oxidized LDL-induced loss of cell viability in accordance with a significant decrease in intracellular NO activity. It suppressed the lipid peroxidation, restored the activities of endogenous antioxidants and decreased the levels of NO and cell apoptosis by preventing the upregulation of poly (ADP-ribose) polymerase-1/nitrotyrosine and inducible NO synthase protein in islet microvascular endothelial cells [14]. GLP-1 also improved the proliferation and differentiation of endothelial progenitor cells by upregulating vascular endothelial growth factor (VEGF) generation [15]. Exendin-4, a GLP-1 agonist, stimulated the proliferation of human coronary artery endothelial cells through eNOS-, PKA- and PI3K/Akt-dependent pathways via the GLP-1 receptor [16]. Exendin-4 also and restored the eNOS-induced ROS production in response to lipotoxicity and protected against lipoapoptosis through PKA-PI3K/Akt-eNOS-p38 MAPK-JNK-dependent pathways via a GLP-1 receptor-dependent mechanism [17]. Liraglutide, another GLP-1 analog, prevented the onset of high glucose-induced endoplasmic reticulum stress in HUVEC [18] and inhibited TNF-α-induced intracellular adhesion molecule (ICAM)-1 and VCAM-1 expression, and these effects were dependent on the GLP-1R [19]. Moreover, we also demonstrated that liraglutide attenuated TNF-α-induced ROS production and increased oxidative stress, and that it increased the expression of anti-oxidant enzymes, including superoxide dismutase-1 and −2, in HUVEC [20].

As DPP-4 inhibitor maintain the plasma level of active GLP-1, sitagliptin augments the protective effects of GLP-1 on the eNOS mRNA level in AGEs-exposed HUVEC with suppressing the receptor for AGE (RAGE) expression and the subsequent ROS generation [21]. Alogliptin induced vascular relaxation via NO and endothelial-derived hyperpolarizing factor-mediated mechanisms, and increased the NO production with increased eNOS phosphorylation in HUVEC, which was not inhibited by GLP-1R antagonist, exendin 9–39, although NO inhibition or endothelial denudation decreased relaxation response. [22]. Therefore, this relaxation by alogliptin may be mediated by GLP-1R-independent mechanisms. Since decreasing the oxidative stress results in a restoration of the eNOS function, all of these phenomena, including the decreased ROS production and increased eNOS expression, in addition to the endothelial repair and promotion of angiogenesis, lead to an amelioration of the endothelial function.

#### The in vivo or ex vivo endothelial function

Exendin-4 significantly increased the NO level, improved the endothelium–dependent vasodilatation and reduced the expression of NF-κB in the aortas isolated from obese rats. This was demonstrated to occur through the cAMP or AMPK-eNOS pathways [23]. Exendin-4 also inhibited the monocyte adhesion and attenuated the formation of atherosclerotic lesions in apolipoprotein E-deficient (ApoE−/−) mice [24]. Liraglutide improved the endothelial function via GLP-1R, increased the eNOS level and reduced the ICAM-1 expression in the aortic endothelium in mice [19]. Liraglutide also inhibited the progression of atherosclerotic plaques, and improve the plaque stability in ApoE−/− mice [25], although the extent of endothelial vasodilatation induced by acetylcholine (ACh) was not changed. Sitagliptin protected the endothelial function of the renal artery in spontaneously hypertensive rats, and exenatide ameliorated the endothelial dysfunction in the renal arteries from hypertensive patients in an *ex vivo* study [26]. On the other hand, Nathanson et al. reported that the endothelial dysfunction induced by triglycerides was not restored by exendin-4 treatment in rat conduit arteries *ex vivo*[27].

### Other possible effects of Incretins on the endothelium, possible effects of incretins on vessels and clinical data

#### Lowering blood pressure

In recent studies, DPP-4 inhibitors and GLP-1 analogs were recognized to lower the systemic blood pressure [28,29]. One of the possible mechanisms underlying this effect is explained by the extraction of Na^+^, because GLP-1 induces natriuresis in humans [30]. Another possibility is the increase in the activity of eNOS described above, because eNOS-knockout mice [31] and the administration of a NOS inhibitor both led to increased blood pressure [32]. Moreover, Kim et al. reported that GLP-1R activation promotes the secretion of atrial natriuretic peptide and a reduction of blood pressure [33].

As DPP-4 cleaves a wide variety of substrates, including stromal cell-derived factor-1 (SDF-1) alpha, which stimulates the bone marrow mobilization of endothelial progenitor cells (EPC) and brain natriuretic peptide (1–32), which is the active form [34], DPP-4 inhibition may repair endothelial cells and improve the cardiac function, thus resulting in an indirect improvement of the endothelial function.

#### Lipid metabolism

DPP-4 inhibitors reduced the serum levels of cholesterol and triglycerides in mice and humans [28,35]. GLP-1 also decreased the lipid absorption *in vivo*[36]. Therefore, incretins may improve the endothelial function by correcting the lipid metabolism. However, exendin-4 did not affect the levels of cholesterol and triglycerides in mice [35].

### Clinical data

Table 2 lists the effects of GLP-1 and GLP-1-related drugs on the endothelial function. Although GLP-1 enhanced the endothelium-dependent and -independent responses to ACh and sodium nitroprusside (SNP) during infusion of insuline, but not during infusion of saline in patients with metabolic syndrome. Furthermore, no changes in the vasodilator reactivity in response to ACh and SNP were seen after GLP-1 was added to insulin and vitamin C or after GLP-1(9–36) was given during hyperinsulinemia [37]. Kelly et al. demonstrated that exendin-4 did not improve the responses of peripheral arterial tonometry compared to metformin in patients with impaired glucose tolerance [38]. In addition, exendin-4 and liraglutide could not improve the FMD responses in obese patients with T2DM [39]. Surprisingly, sitagliptin and alogliptin actually led to a deterioration in the FMD responses in patients with T2DM [40]. Since the DPP-4 inhibitors inhibited DPP-4 activity and increased the level of GLP-1, in addition to lowering the glucose level, in this study, it is clear that the DPP-4 inhibitors worked efficiently. The authors suggested that GLP-1(9–36), a metabolite of GLP-1 cleaved by the DPP-4 inhibitor, was thought to be inactive, but possessed a vasodilatory effect *ex vivo* which was mediated by NO [6]. However, sitagliptin, alogliptin and vildagliptin also improved the endothelial function in other studies. Therefore, it is hard to simply conclude whether the discrepancy was due to the differences in the drugs or the inhibition of GLP-1 (9–36).

**Table 2:** Clinical evaluation of endothelial function

**Author**	**Subjects**	**n**	**Endothelial function**	**Medication**	**Control**	**Duration**	**Result**
**Basu A et al.**[41]	**T2DM**	**29**	**Strain-gauged plethysmography**	**GLP-1**	**Placebo**	**240 min**	**Improved**
**Kubota Y et al.**[42]	**T2DM**	**40**	**FMD**	**Sitagliptin**	**None**	**12 w**	**Improved**
**Nystrom T et al.**[43]	**T2DM**	**12**	**FMD**	**GLP-1**	**Saline**	**115 min**	**Improved**
**Koska J et al.**[44]	**T2DM**	**28**	**PAT**	**Exendin-4**	**Saline**	**210 min**	**Improved**
**Noda Y et al.**[45]	**Healty volunteer**	**10**	**FMD**	**Alogliptin**	**Placebo**	**1 w**	**Improved**
**van Poppel PC et al.**[46]	**T2DM**	**16**	**Strain-gauged plethysmography**	**Vildagliptin**	**Acarbose**	**4 w**	**Improved**
**Ceriello A et al.**[47]	**T2DM**	**28**	**FMD**	**GLP-1**	**Saline**	**2 h**	**Improved**
**Irace C et al.**[48]	**T2DM**	**20**	**FMD**	**Exendin-4**	**Glimepiride**	**16w**	**Improved**
**Tesauro M et al.**[37]	**Metabolic syndrome**	**10 (5/5)**	**Strain-gauged plethysmography**	**GLP-1**	**Saline**	**30 min**	**Improved (conditional)**
**Kelly AS et al.**[38]	**IGT**	**50**	**PAT**	**Exendin-4**	**Metformin**	**6 m**	**No change**
**Hopkins ND, et al.**[39]	**Obese T2DM**	**11**	**FMD**	**Exendin-4 (n = 9) Liragrutide (n = 2)**	**None**	**6 m**	**No change**
**Ayaori M et al.**[40]	**T2DM**	**13 ~ 20,22**	**FMD**	**Sitagliptin/alogliptin**	**Voglibose**	**6 w**	**Worsened**

T2DM: type2 diabetes mellitus, GLP-1: glucagon-like peptide-1, FMD: flow-mediated dilation, IGT: impaired glucose tolerance, PAT: Peripheral arterial tonometry.

On the other hand, nine studies previously reported that the endothelial function was improved by using GLP-1 and GLP-1-related drugs [37,41-48]. This effect is still being debated, because all of these studies included a small number (< 50) of patients, and almost all were non-randomized trials. Therefore, a large-scaled randomized trial will be necessary to clearly define the impact of these agents on the endothelial function.

We designed and are performing an ongoing multicenter randomized prospective study to evaluate the effects of DPP-4 inhibition on carotid atherosclerosis by measuring the carotid intima-media thickness (PROLOGUE trial: UMIN000004490). In this trial, the effects of a DPP-4 inhibitor on the FMD will be analyzed as part of a subgroup of the study. The result of this study may answer the questions remaining regarding the effects of DDP-4 inhibition.

### Do incretins improve the prognosis and mortality from cardiovascular disease?

Recently, the results of cardiovascular safety trials of type 2 diabetes drugs, EXAMINE trial with alogliptin and SAVOR-TIMI 53 trial with saxagliptin, were reported [49,50]. These two studies found no effect on the risk of fatal or non-fatal cardiac events and no increases in the risk of pancreatitis or pancreatic cancer. The results were disappointing because the studies did not demonstrate any cardiovascular protective benefits of DPP-4 inhibitors. There are a few limitations. First, the follow-up period was too short to evaluate the incidence of cardiovascular events, because the effects of drugs in fighting pro-atherosclerotic processes in patients with T2DM requires more than 10 years. Second, the relatively small HbA1c-lowering effects of saxagliptin and alogliptin observed in both trials, averaging only 0.3 to 0.4 percentage points. It may influence the final results. Further sub-analyses and other ongoing trials need to be waited.

We need to admonish against jumping illogically.

### Additional features and limitations

The blood vessels modulate vascular tone and blood flow by constricting or relaxing in response to physical, neurological and chemical stimuli. Endothelial dysfunction is recognized as a major factor in the development of atherosclerosis and FMD is designated as an endothelium-dependent process that reflects the relaxation of a conduit artery (brachial, radial, and femoral) when exposed to increased blood flow and shear stress and recognized as useful tool for the assessment of endothelial function in different clinical and research populations. Recently FMD is recognized as an independent predictor of future cardiac events [51,52].

It is a simple and widely available method, however, there are several caveats due to the potential technical variations [53], these include:

1) An acceptable reproducibility is a mean difference of 2% to 3% in the FMD over time. To measure the FMD precisely, 100 independent supervised scans and measurements are required according to a previous report.

2) At least 40 to 60 patients in a parallel-group study are needed because of the fluctuation of the data.

3) The diameters of small arteries at baseline appear to dilate more than those of larger arteries, and repeated measurements of the diameter at baseline must be the same. Therefore, the precise vessel diameters before and after reactive hyperemia (mean baseline and peak deflation diameters) have to be addressed in addition to the % changes of the FMD.

4) In multicenter studies, the methods used for the measurement should be unified, and a core laboratory should be used to analyze the FMD data to minimize the variations among the different centers.

Measurement errors are present in all of the data because of the biological variations and methodological limitations described above. Therefore, the evaluations must be performed carefully and precisely. Guidelines for measuring the FMD have been published [53-55], and the Japanese Circulation Society will announce new guidelines in 2014. Therefore, it is necessary to recognize the limitations and the features of FMD and to measure the FMD as precisely as possible in compliance with the guidelines.

In addition to atherosclerosis, incretins may prevent myocardial ischemia-reperfusion (I/R) injury. Sitagliptin or vildagliptin for two weeks reduced infarct size after myocardial I/R injury through the GLP-1 receptor-PKA pathway, in a glucose-dependent manner in rat in ex vivo [56] In addition, exendin-4 could attenuate myocardial I/R injury which may be associated with inhibiting the expression of high mobility group box 1 in ra in vivo [57]. As myocardial ischemia-reperfusion is critical for the patients with coronary artery disease or acute coronary syndrome who underwent percutaneous coronary intervention, incretin-therapy may have beneficial effects beyond glycemic control.

Finally, we would like to add that this review is not a systematic review or meta-analysis based on the PRISMA guidelines [58].

## Conclusions

Atherosclerosis and the subsequent cardiovascular disease are fatal, and early prevention of cardiovascular complications by ensuring strict glucose control is essential for patients with T2DM. The current findings add to a growing body of evidence that suggests that we might be entering a new era of cardiovascular diabetology with new anti-diabetic drugs for now. On the other hand, several investigations contradicted the beneficial effects of incretin on the vasculature. Therefore, as a next step, large-scaled randomized, prospective, clinical studies and their subanalyses will provide the evidence whether incretin therapy provide clinical benefits of vascular protection beyond glycemic control for patients with T2DM who are at risk of cardiovascular disease.

## Abbreviations

T2DM: Type 2 diabetes mellitus; GLP-1: Glucagon-like peptide-1; GIP: Insulinotropic polypeptide; GLP-1R: GLP-1 receptor; DPP: Dipeptidyl peptidase; CVD: Cardiovascular disease; NO: Nitric oxide; FMD: Flow-mediated dilation; ROS: Reactive oxygen species; AGE: Advanced glycation end product; VCAM: Vascular cell adhesion molecule; HUVEC: Human umbilical vein endothelial cells; eNOS: Endothelial NO synthase; VEGF: Vascular endothelial growth factor; ICAM: Intercellular adhesion molecule; RAGE: The receptor for AGE; ApoE-/-: apolipoprotein E deficient; SDF: Stromal cell-derived factor; ACh: Acetylcholine; SNP: Sodium nitroprusside.

## Competing interests

The authors declare that they have no competing interests.

## Authors’ contributions

All authors contributed to conception and design, drafting the article, revising the article critically, and final approval of the version to be published.
